# mRNA Vaccination: An Outlook on Innate Sensing and Adaptive Immune Responses

**DOI:** 10.3390/v16091404

**Published:** 2024-09-01

**Authors:** Janan Shoja Doost, Fatemeh Fazel, Nitish Boodhoo, Shayan Sharif

**Affiliations:** Department of Pathobiology, Ontario Veterinary College, University of Guelph, Guelph, ON N1G 2W1, Canada; jshojado@uoguelph.ca (J.S.D.); ffazel@uoguelph.ca (F.F.); boodhoon@uoguelph.ca (N.B.)

**Keywords:** mRNA vaccine, innate sensing, adaptive responses, adjuvanticity

## Abstract

Vaccination has led to significant dismantling of infectious diseases worldwide. Since the dawn of the SARS-CoV-2 pandemic, there has been increased popularity in the usage and study of the mRNA vaccine platform. Here, we highlight fundamental knowledge on mRNA vaccine pharmacology, followed by the immunity conferred by innate sensing and adaptive responses resulting from exposure to the mRNA vaccine construct and encapsulation materials. A better understanding of these immune mechanisms will shed light on further improvements in mRNA vaccine design, aiming to improve efficiency and optimize immune responses upon inoculation.

## 1. Introduction

Immunization is the most potent measure in curtailing pathogen transmission in humans and animals, and technologies for the establishment of optimal and effective vaccines are ever-advancing [1]. Usage of exogenously produced nucleic acids as drug was initially conceptualized by Wolff and colleagues over three decades ago, showing that direct injection of in vitro-transcribed (IVT) mRNA or plasmid DNA (pDNA) into the skeletal muscle of mice resulted in expression of the encoded protein within the injected muscle [2]. Subsequent studies examined IVT mRNA in its application to protein substitution [3] and vaccination approaches [4,5,6,7] for infectious diseases. Further research highlighted incorporation of modified nucleotides into the mRNA design leading to enhanced stability and translatability [8], with this body of knowledge being awarded the 2023 Nobel Prize in Physiology or Medicine [9].

With the onset of the SARS-CoV-2 pandemic, mRNA-based vaccines proved remarkably successful, showing high efficacy against symptomatic infection caused by different strains of this virus [10], and effective induction of host immunity post infection [11]. To better understand mRNA vaccine efficacy, it is crucial to investigate the role of various elements used in the vaccine design and their impact on immune responses post vaccination. Thus, in this review, we discuss the current state of knowledge on mRNA vaccine pharmacology, detail the mechanisms of mRNA and encapsulation material innate sensing as well as the adaptive immunity conferred, with this knowledge paving the path for vaccine optimization and efficient induction of immune responses to benefit the host.

## 2. mRNA Vaccine Fundamental Pharmacology

### 2.1. mRNA Construct

mRNA vaccine production capitalizes on the principle of transcription from DNA to protein translation via cytoplasmic ribosomes using the IVT method. Linear mRNA, commonly known as the non-replicating mRNA (NRM), is a conventional construct representing endogenous eukaryotic mRNA constituted of a 5′ cap, a 5′ untranslated region (5′ UTR), an open reading frame (ORF), a 3′ untranslated region (3′ UTR), and a 3′ poly A tail, all arranged in a sequence from the 5′ end to the 3′ end [12]. The 5′ cap structure facilitates binding of the eukaryotic translation initiation factor 4E (eIF4E) to the mRNA’s 5′ cap in order to initiate translation [13]. UTRs are essential for mRNA stability and protein interaction to ensure efficient translation—recently, a predictive model was developed to design novel UTR sequences and investigate mRNA translation efficiency by using deep learning technology [14]. The ORF represents the encoded genetic information for the target protein, and translation effectiveness by ORF manipulation can be enhanced through codon optimization [15]. The poly A tail assists with mRNA stability [16] and the initiation of translation by recruiting factors 4G and 4E [17]. It has also been shown that the sequence length of the poly A tail majorly impacts translational efficacy [18,19]. Together with these translation factors and the 5′ cap, the poly A tail makes a closed loop construct, protecting the structure from enzymatic degradation and allowing for re-entry into the ribosome [20]. Moreover, similar to the NRM vaccine construct, the self-amplifying mRNA (SAM) design is composed of similar transcriptional elements and additionally includes an RNA virus-derived replicase to facilitate rapid amplification of transcripts in host cells using a lower mRNA vaccine dose [21], inducing comparable immune responses to the NRM vaccine [22]. The self-amplification quality of SAM leads to the production of double-stranded RNA (dsRNA), triggering antigen-specific immune responses by binding to pattern recognition receptors (PRRs) and contributing to vaccine adjuvanticity [23]. Thus, current mRNA constructs allow for stable transcription of mRNA in vitro with implications for immune response induction post translation.

### 2.2. mRNA Modifications

While fundamental mRNA designs allow for production of mRNA in vitro, various methodologies have been introduced to improve stability and efficiency of the encoded-protein translation. As discussed earlier, Nobel-prize winning work by Karikó and colleagues highlighted the value of codon optimization through incorporation of pseudouridines (ψ), showing enhanced protein translatability and diminished immunogenicity in vivo [8]. Pseudouridine incorporation also reduced activation of protein kinase R (PKR) and prevented repression of protein translation [24]. Conversely, sequence-engineered mRNA successfully enabled protein therapy in large animals without chemical nucleotide modifications [25].

mRNA purification via high-performance lipid chromatography (HPLC) has also been shown to improve translatability by removing dsRNA and eliminating immune activation [26]. Fast protein liquid chromatography (FPLC) has also been proven to enhance protein translation, as human immunodeficiency virus (HIV)-1-infected mice treated with purified m1ψ-modified mRNAs showed increased production of antibodies [27]. RNAse III has also been shown to be effective in purifying mRNA by removing dsRNA and improving mRNA quality, thereby enhancing immune responses through robust T-cell activity [28]. In addition, a LinearDesign algorithm efficiently found an ideal mRNA sequence for the SARS-CoV-2 spike protein within 11 min, all while simultaneously searching for sequences with enhanced stability and codon optimization [29]. Thus, modification and purification of mRNA constructs may allow for efficient translatability and enhanced induction of immune responses.

### 2.3. mRNA Encapsulation

Various challenges may arise with mRNA transport and delivery to target cells due to their innate physical and biochemical properties. To facilitate diffusion across plasma membranes, mRNAs must overcome their negative charge and hydrophilicity. An early study in 1989 showed successful transfection of mRNA using a cationic liposome structure [30]. Recent advances in development of an ionizable lipid nanoparticle (LNP) platform show efficient mRNA encapsulation, high cellular transfectability, as well as minimal impacts on cell viability and adverse immune responses [31]. Due to their size and surface charge characteristics, cells internalize LNPs through endocytosis, and lipid ionizations under low-pH conditions facilitate their escape from endosomes, thereby enabling release of the cargo into the cytoplasm [32]. Optimization of LNP size is crucial for mRNA encapsulation and improving half-life, with smaller LNPs being less likely to be taken up by phagocytes [33]. For pharmaceutical applications, particularly for parenteral administration, particles are recommended to be 100 nm or smaller for optimal uptake [34,35]. Furthermore, LNP surface charge is primarily determined by the ionic charge of the lipid head groups and is typically expressed through zeta potentials, with potentials less than −30 mV or greater than 30 mV ensuring interparticle repulsion and stable suspensions [36]. While such elements impact LNP stability to favor usage of these constructs as mRNA delivery vehicles, other factors such as surface composition and internal constituents further influence their cellular uptake, stability, and half-life. LNP interaction with extracellular proteins, such as apolipoprotein E (ApoE), allows for redistribution of lipids between the LNP shell and core to alter its internal structure, ultimately enabling mRNA release [37]. Composition-wise, LNPs typically contain a helper lipid to enhance cell binding, cholesterol to cushion the lipids, and polyethylene glycol (PEG) to minimize recognition by serum proteins and clearance by the reticuloendothelial system, with the proportions of these components significantly influencing LNP efficacy [32]. Covalent binding of PEG to the LNP construct (PEGylation) effectively creates a steric barrier to reduce the binding of blood proteins and decrease clearance by macrophages, thus improving bioavailability [38,39]. Additionally, PEGylation impacts overall LNP stability and half-life, with factors such as PEG length and concentration playing a role in preventing LNP aggregation [40]. Recent work on modifications to the PEG construct via fluorination has demonstrated enhanced mRNA delivery through LNPs, with improved expression of the delivered mRNA in dendritic and tumor cells as well as effective cellular uptake and endosomal escape even when administered in vivo [41]. In a practical setting, LNPs have been successfully used as the delivery system in the Pfizer/BioNTech and Moderna COVID-19 mRNA vaccines, developed rapidly and proven highly effective in controlling disease [42]. The LNP composition of both vaccines is similar, as they contain an ionizable lipid that is positively charged at low pH for RNA binding and neutral at physiological pH to minimize toxicity and aid in payload release. Additionally, they include a PEGylated lipid to reduce opsonization and clearance by phagocytes. Furthermore, the phospholipid distearoylphosphatidylcholine (DSPC) and cholesterol assist in packing the mRNA cargo into LNPs. The molar ratios of cationic lipid, PEG-lipid, cholesterol, and DSPC are 46.3:1.6:42.7:9.4 and 50:1.5:38.5:10 for the Pfizer and Moderna LNP constructs, respectively [43]. These nanoparticles are 80–100 nm in diameter [44] and contain about 100 mRNA molecules each [45]. Thus, with size, charge, and overall composition impacting their stability, half-life, and cellular uptake, LNPs are appropriate vehicles for the delivery of nucleic acid cargo in the context of mRNA vaccines.

Chitosan is another compound used for encapsulation of mRNA materials. Sourced from chitin, a prevalent polysaccharide in nature, chitosan is produced naturally. While it is less abundant than chitin and exists in specific fungi species, neither chitosan nor chitin is present in mammals [46]. The absence of chitin or chitosan within mammalian cells suggests that these polymers could be recognized by the innate immune system, making them potential targets for innate sensing. Both particles are easily engulfed through phagocytosis, suggesting a receptor-mediated mechanism for this function [47]. More will be discussed on innate and adaptive immune sensing of chitosan-encapsulated compounds in subsequent sections.

## 3. Innate Sensing of mRNA and Encapsulation Compounds

### 3.1. Role of Innate Elements in mRNA Sensing

Various types of RNA have shown to initiate signaling of PRRs. dsRNA, often observed as a viral constituent, has shown to induce Toll-like receptor (TLR)3 signaling [48]. To assess TLR3 activation by RNA, a human embryonic kidney 293 cell line expressing TLR3 and a luciferase reporter dependent on nuclear factor kappa B (NF-kB) were utilized [49]. IVT RNA exposure led to TLR3-dependent elevation in luciferase activity and interleukin (IL)-8 secretion [49]. IVT mRNA also dose-dependently activated NF-kB through TLR3, mediated by tyrosine phosphorylation [49]. Additionally, both natural and 2′-fluoro-substituted IVT mRNA prompted the expression of TLR3, interferon regulatory factor 1 (IRF1), tumor necrosis factor alpha (TNF-α), and interleukin-1 receptor-associated kinase (IRAK)-M genes in human dendritic cells (DCs) [49]. The treatment of DCs with mRNA resulted in the expression of markers associated with activation, and this maturation was impeded by a specific antibody that antagonizes TLR3 [49]. Retinoic acid-inducible gene I (RIG-I) is another innate element sensing dsRNA, for which confirmational alterations are hindered through usage of nucleotide-modified RNA, thereby preventing initiation of innate immune signaling [50]. RIG-I can recognize m7GpppN cap0 structure in the 5′ cap of the mRNA construct [51,52] and induce downstream proinflammatory cascades; thus, a cap1 structure (m7GpppNm), generated through treatment of cap0-mRNA with 2′-O-methyltransferase, is characterized by methylation on the first nucleotide near the cap to facilitate its recognition as “self” [53]. Next, single-stranded RNA (ssRNA) was shown to activate mouse TLR7, showing its role in innate anti-viral responses [54]. Also, it has been demonstrated that murine TLR7 and human TLR8 are responsible for species-specific identification of guanosine and uridine-rich ssRNA [55]. Specifically, TLR7 interacts concurrently with guanosine and the tri-ribonucleotide UUU [56], and TLR8 recognizes uridine and the di-nucleotide UG [57]. Moreover, both TLR7 and myeloid differentiation primary response 88 (MyD88) were shown to be required for recognition of ssRNA, as TLR7^−/−^ and MyD88^−/−^ mice showed reduced responsiveness to vesicular stomatitis virus by decreased production of interferon (IFN)-α in vivo [58]. Melanoma differentiation-associated protein 5 (MDA5) is another innate element that typically senses long dsRNA molecules or ssRNA with secondary structures [59]. MDA5 and RIG-I have shown to be structurally and functionally homologous, as IFN-α and IFN-β genes were specifically inhibited when RIG-I or MDA5 were knocked down by siRNA [60]. Thus, various innate elements are capable of recognizing RNA of different forms.

### 3.2. mRNA Modifications in Innate Sensing

**Figure 1:**
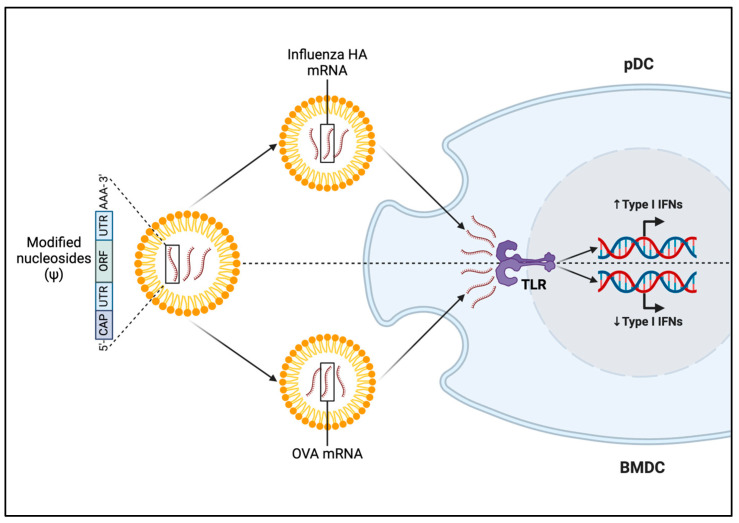
Innate immune responses resulting from mRNA nucleoside modification are disease- and condition-dependent. LNP-encapsulated mRNA with modified nucleosides encoding influenza H10 HA induced type I IFN via TLR7 in plasmacytoid DCs [64]. Yet, m1Ψ modification in LNP-encapsulated OVA mRNA reduced type I IFN production in BMDCs in a melanoma context [65].

As discussed previously, mRNA modifications facilitate translation processes and induction of immune responses post treatment. In a landmark study by Karikó and colleagues, the presence of modified nucleosides such as m5C, m6A, m5U, s2U, or pseudouridine was shown to nullify the activity of TLR3, TLR7, and TLR8, and DCs exposed to modified RNA exhibited notably reduced expression of cytokines and activation markers compared to those treated with unmodified RNA [61]. Since then, many studies have experimented with modified nucleotides and their effects in various organ systems, cell types and conditions (Figure 1). Liposome-encapsulated mRNA has been shown to induce IFN-α production in mice in vivo through a TLR7-dependent mechanism [62]. Plasmacytoid DCs (pDCs) have long been shown to express TLR7 and secrete high levels of type I IFNs, such as IFN-α, important for promoting antiviral functions and priming adaptive immunity [63]. Thus, to further look into the protective role of pDCs through TLR7 signaling and type I IFN production in an mRNA vaccine setting, pDCs were examined in vitro for their IFN-α secretion abilities after administration of a nucleotide-modified LNP-encapsulated NRM vaccine construct against influenza H10 hemagglutinin (HA) [64]. Compared to empty LNP, the modified mRNA/LNP construct triggered noticeable IFN-α production in pDCs, yet this effect was less pronounced compared to actions of the synthetic TLR7/8 agonist, R848 [64]. Nucleotide modification of the mRNA construct has also been shown to impact outcomes in a cancer setting. The immunogenicity and anti-tumor responses of mRNA-encoding tumor antigens with different degrees of N1-methylpseudouridine (m1Ψ) modification were examined in a B16 melanoma model [65]. With increasing percentages of m1Ψ modification in an ovalbumin (OVA)-encoding mRNA encapsulated within LNPs, there was significant decrease in type I IFN levels and reduced DC maturation [65]. Contrary to prior knowledge about the impact of mRNA modification in improving disease outcomes, the unmodified mRNA construct significantly reduced tumor growth, prolonged survival, and led to an increase in intra-tumoral cluster-of-differentiation (CD)40+ DCs compared to OVA-LNP with m1Ψ modification in the B16-OVA murine melanoma model [65]. Thus, mRNA modifications do play a role in modulating immune responses post vaccination, albeit with such effects being disease- and condition-dependent (Figure 1).

Modifications in UTRs of mRNA sequences were also shown to impact immunity post vaccination. An LNP-encapsulated mRNA vaccine construct made against the Varicella zoster virus utilized the addition of different UTRs to a double-mutated region in the sequence of glycoprotein E (gE-M), demonstrating strong humoral and cell-mediated immunity through surges in the secretion of IL-2 and IFN-γ by splenocytes when the construct included gE m sequences in conjunction with Pfizer/BioNTech’s BNT162b2 UTRs [66]. Other types of sequence-wise modifications have also been shown to impact innate immune responses post vaccination. In a hepatitis C virus (HCV) setting, LNP-encapsulated mRNA vaccines were conjugated to viral ectodomains of E1, E2, or a modified E2 with reduced CD81 binding and an inserted N-linked glycosylation site [67]. Co-immunization with soluble E1 and E2 has been shown to significantly dampen T helper (Th)1-type cytokine production while sequence-modified soluble E2 led to significant increase in Th1 responses via the release of IL-2 and IFN-γ, and a decrease in Th2 type responses through attenuated IL-4 and IL-10 levels [67]. Thus, different sequence modifications in the mRNA construct may lead to altered innate sensing, leading to variable condition-dependent outcomes.

### 3.3. Encapsulation Material in Innate Sensing

Moreover, LNP composition has been shown to be important in the activation degree of PRRs. An LNP-encapsulated mRNA vaccine with a composition of a 12-carbon-tailed cationic lipid with PEG was rendered ineffective through pharmacological modulation of TLR4 activity using small molecule inhibitor TAK-242 in bone marrow-derived dendritic cells (BMDCs), showing reduced transcription of IL-1β, IL-6, and IL-2p40 [68]. In another study, an LNP-encapsulated modified NRM vaccine construct against influenza H10 HA was shown to rapidly induce expression of IL-1β, MyD88, pentraxin-related protein 3 (PTX3), and NOD-like receptor family pyrin domain-containing 3 (NLRP3), increase infiltration of neutrophils, and upregulate CD80 and CD86 co-stimulatory receptors of monocytes and DCs in the intramuscular and intradermal vaccine-injection sites and draining the lymph nodes of rhesus macaques [64]. Moreover, to determine anti-tumor efficacy of a simplistic C1 LNP-encapsulated mRNA vaccine, the innate impacts of the construct were evaluated against BMDCs, and it was shown that LNP on its own, similar to LNP-encapsulated mRNA, was able to increase CD80 and CD86 surface markers, as well as upregulate expression of proinflammatory cytokines IL-1β, IL-6, and IL-12p40 and type I IFNs such as IFN-α1, IFN-α4, and IFN-β1 [68]. Furthermore, the efficacy of nucleotide-modified mRNA has been shown to be affected by the specific chemistry of the LNP delivery system used. Replacing uridine with N1-methylpseudouridine in an mRNA vaccine for influenza hemagglutinin notably reduced the production of innate chemokines and cytokines such as IL-1β, IL-6, IL-8, IL-17, IFN-α2, C-X-C motif chemokine ligand (CXCL)11, C-C motif chemokine ligand (CCL)2, TNF-α, and positively affected the generation of effective antibody levels in mice and macaques when using MC3 or KC2 LNPs for delivery [69]. However, this modification had a relatively small effect on the antibody levels achieved using L319 LNPs, thus emphasizing that the effectiveness of modified mRNA differs depending on the composition of the LNPs used for delivery [69].

Chitosan encapsulation methods have also been shown to elicit innate responses. When examined by itself, chitosan was shown to induce innate responses by increasing mRNA expression of TNF-α and viperin, an interferon stimulating gene, in the spleen and mesonephron of *Siniperca chuatsi* fish infected with infectious spleen and kidney necrosis virus (ISKNV), while also preventing virus replication and improving antiviral functions [70]. In addition, chitosan was also demonstrated to strongly induce the NLRP3 inflammasome, leading to elevated production of one of the inflammasome’s functional cytokines, IL-1β [47]. Chitosan nanocarriers also induced expression of proinflammatory cytokines such as IL-6, TNF-α, and IFN-γ in human peripheral blood mononuclear cells (PBMCs) [71]. Chitosan nanocarriers engineered with tri-mannose ligands were able to remodel macrophage response against infection with *M. tuberculosis* in a cell culture model of human monocytes as shown through RNA sequencing experiments, in which the engineered chitosan nanocarrier significantly upregulated genes involved in cytokine–cytokine receptor interactions, TNF and NF-kB signaling, chemokine signaling, and the TLR/NLR pathways [72]. Chitosan was also able to significantly reduce LPS-induced expression of TNF-α, IL-6, IL-10, and IL-12 in human PBMCs, as well as decrease expression of TLR2 and TLR4 [73]. Regarding encapsulation of vaccination material with chitosan, there was enhanced expression of TLR1, TLR4, TLR5, and TLR7 at 7 days post oral inoculation of broiler chickens with an mRNA vaccine constructed against the outer membrane protein and flagellin of *Salmonella enterica* serovar Enteritidis (SE) and encapsulated with chitosan nanoparticles [74]. The same team of researchers further investigated the impacts of conjugating the chitosan-encapsulated vaccine with mannose, and observed a significant increase in expression of TLR3 and TLR7, as well as IL-1β, TNF-α, IL-10, transforming growth factor beta (TGF-β) in the cecal tonsils of broiler chickens compared to the control non-encapsulated vaccines [75]. In a separate study, while examining the efficacy of chitosan microparticle-encapsulated vaccine against oral fowl typhoid, increased levels of IFN-γ were reported in the spleen post vaccination and there was 100% protection when chicks were challenged with the 85 kb virulence plasmid SG9, although these effects were not statistically significant when compared to a conventional vaccine administered subcutaneously [76]. The molecular weight of chitosan particles was also shown to impact innate immunogenicity in vitro—low molecular weight (LMW; 50–190 kDa) and high molecular weight (HMW; 310–375 kDa) chitosan particles were examined for their effects in BMDCs [77]. Both chitosan compounds were able to upregulate expression of IL-6, IFN-β1, and CXCL-10 24 h post treatment [77]. It was shown that HMW chitosan would induce such effects by activating the interferon regulatory pathway as early as 18 h post treatment, followed by subsequent significant effects of the LMW chitosan at later timepoints [77]. Recently, IL-7 from Tibetan pigs was used to construct a recombinant eukaryotic plasmid (VRTPIL-7). This recombinant plasmid was then encapsulated with chitosan nanoparticles and conjugated against a rabies vaccine construct in mice, showing elevated mRNA expression of TLR1, TLR4, TLR6, TLR9, IL-1, IL-2, IL-4, IL-6, IL-7, IL-23, and TGF-β post inoculation [78].

### 3.4. Role of Innate Elements in mRNA Vaccine Adjuvanticity and Functionality

**Figure 2:**
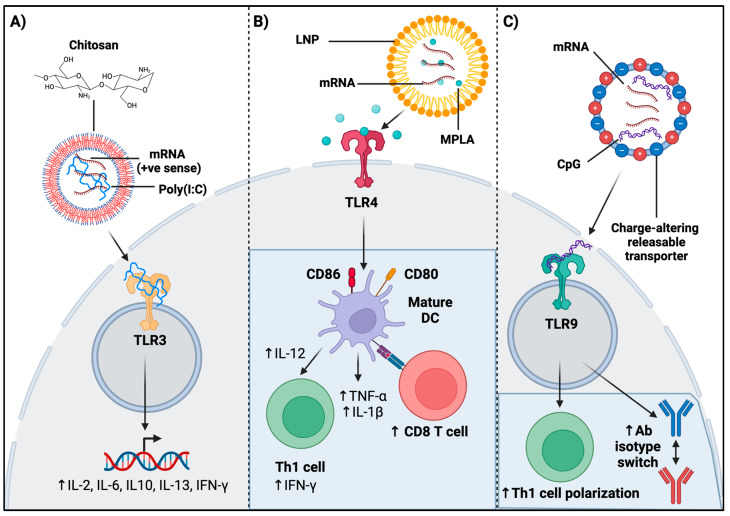
mRNA vaccine adjuvanticity with TLR ligands impacts downstream innate and adaptive immune responses. (**A**) Chitosan-coated swine influenza vaccine with poly(I:C) increased IL-2, IL-10, IL-13, IL-6, and IFN-γ in pig tracheobronchial lymph nodes post inoculation [79]. (**B**) LNP-encapsulated OVA mRNA with MPLA induced DC maturation genes (CD80, CD86) in BMDCs, leading to enhanced Th1 differentiation, IFN-γ production, increased TNF-α and IL-1β, and augmented infiltration of cytotoxic CD8+ T cells, slowing melanoma growth [80]. (**C**) An mRNA vaccine against SARS-CoV-2 RBD, utilizing charge-altering releasable transporters, showed CpG addition enhanced anti-RBD antibodies, early isotype switching, increased Th1 polarization, and CD4+/CD44high/TNF-α+ expression [82].

TLR agonism has been utilized for tissue-specific delivery of mRNA vaccination and adjuvanticity functions. Conjugation of a chitosan-coated vaccine against swine influenza virus antigen with TLR3 ligand, poly(I:C), was shown to significantly increase IL-2, IL-10, IL-13, as well as IL-6 and IFN-γ in tracheobronchial lymph nodes of pigs post inoculation (Figure 2A) [79]. Moreover, a study by Pan and colleagues investigated development of a potent anti-tumor vaccine co-loaded with stearic acid-doped lipid nanoparticles (sLNPs), OVA-encoding mRNA, and a TLR4 agonist, monophosphoryl lipid A (MPLA) [80]. The results from RNA-seq analysis of BMDCs treated with sLNPs-OVA/MPLA or sLNPs-OVA showed that, compared to sLNPs-OVA treatment, sLNPs-OVA/MPLA treatment enriched differentially expressed genes in immune stimulating, antigen processing, and antigen presentation-associated signaling pathways. Gene signature analysis revealed that Th1/Th2 cytokines, chemokines, DC maturation, and antigen presentation-related key genes in BMDCs were upregulated compared to those in sLNPs-OVA treated BMDCs (Figure 2B) [80]. It was shown that sLNPs-OVA/MPLA offered more immunogenicity compared to LNP alone and facilitated tissue-specific mRNA expression in the spleen after intravenous injection, with significant upregulation of proinflammatory cytokines such as TNF-α, IL-1β, and IL-12, and DC maturation genes, such as CD80 and CD86 (Figure 2B) [80]. Furthermore, co-delivery of TLR4 ligand MPLA alongside 5meC nucleotide-modified mRNA of a truncated OVA encapsulated in a lipoplex led to significant increased expression of CD11c+ splenocytes [81]. Also, there was a notable release of IFN-γ, IL-6, and CCL-2 with this adjuvanted mRNA vaccine construct 12 h post immunization, which was comparable to high cytokine levels 6 h post treatment with non-adjuvanted constructs, demonstrating that TLR4 agonist adjuvanticity delayed immune activation and inflammatory cytokine response [81].

In addition, conjugation of an LNP-encapsulated mRNA vaccine against the SARS-CoV-2 spike glycoprotein with stimulator of interferon genes (STING) agonist-derived amino lipids (SALs) was shown to be the most effective in mRNA deliverance to DCs and led to upregulation of CXCL1, CXCL9, CCL4, and granulocyte-colony stimulating factor, but not IL-6, post the immunization of mice [83].

### 3.5. Functional Overlap in Innate Sensing of mRNA Vaccines

Despite the role of innate sensor stimulation in vaccine adjuvanticity, there is functional overlap and elements can still induce immunogenicity through a compromise in innate signaling. The efficacy of the SARS-CoV-2 Pfizer-BioNTech BNT162b2 vaccine regarding antibody and CD8+ T-cell responses did not appear to diminish in the absence of TLR2, TLR4, TLR5, TLR7, or STING [84]. This suggests that these pivotal detectors of nucleic acids and microbial lipids might not be necessary for eliciting a robust immune reaction to this particular vaccine [84]. TLR3 deficiency resulted in a moderate (2.4-fold) decrease in neutralizing antibody titers, although there was no impact on spike-binding IgG, indicating a potential minor role in enhancing immune response [84]. Additionally, mice lacking MDA5 exhibited a substantial reduction in the frequency of antigen-specific CD8+ T cells but only a mild effect on antibody responses post vaccination [84]. This hints at the possibility that the MDA5-IFN-α signaling pathway might contribute to specific aspects of the immune response to nucleoside-modified mRNA-LNP vaccines, particularly the robust CD8+ T-cell response observed with the BNT162b2 vaccine, yet does not significantly influence antibody-mediated protection [84]. When examining influenza and SARS-CoV-2 immune responses after treatment with LNP-encapsulated mRNA vaccines against PR8 HA mRNA-LNP and SARS-CoV-2 receptor binding domain (RBD) mRNA-LNP, respectively, it was shown that compensatory factors could still help with eliciting appropriate immune responses even if elements of innate immunity were rendered dysfunctional [84]. On this note, Langerhans cells and conventional type 1 DCs (cDC1s) played redundant roles in adaptive protection against influenza virus and SARS-CoV-2, as their genetic ablation led to similar induction of T helper follicular cells, germinal B cells, and plasma cells [85]. In addition, the ablation of neutrophils did not impact the development of PR8 HA-specific T helper follicular cells or B-cell responses, and the slight decline in serum anti-HA IgG levels in the absence of neutrophils was not deemed as significant [85]. Thus, adjuvanticity through the activation of innate sensors has been shown to modulate immune responses post vaccination, although some evidence exists on the overlapping functions of some PRRs involved in the innate sensing of mRNA vaccines.

### 3.6. Novel Approaches in Evaluating Efficacy of mRNA Vaccine and Encapsulation Material Innate Sensing

Bioinformatic tools have been utilized to predict interactions of innate immune elements with mRNA vaccines. To evaluate the binding affinities of a predicted universal SARS-CoV-2 vaccine to TLR3 and TLR9, the tertiary structure of the predicted vaccine construct was docked against the TLRs, showing a high affinity by a binding score of −143.99 for TLR3 and 0 for TLR9 [86]. Also, an immune simulation was conducted in silico using the C-ImmSim platform, showing activation of CD4 T helper lymphocytes, CD8+ lymphocytes, and B and plasma cells, with predicted IgM and IgG secretion activities [86].

Moreover, a reverse vaccinology approach was used to optimize the design of an HIV mRNA vaccine [87]. Five HIV-surface proteins from the existing literature were used to screen potential epitopes and formulate an mRNA vaccine using diverse immunological-informatics [87]. The final vaccine consisted of 31 epitopes, a TLR4 agonist known as RpfE functioning as an adjuvant, secretion enhancers, structures for subcellular trafficking, and connecting elements [87]. Docking experiments with TLR4 and TLR3 demonstrated substantial interactions at −11.9 kcal/mol and −18.2 kcal/mol, respectively [87]. Molecular dynamics simulations confirmed the vaccine’s stability, and codon optimization was executed to ensure successful translation of the designed mRNA construct within the host [87]. The same group also utilized a similar methodology to predict an mRNA vaccine design against *Serratia marcescens*, a Gram-negative bacterium [88]. Codon optimization was conducted in silico to guarantee the mRNA’s successful loading in the cytoplasm of a human host and its efficient translation [88]. Additionally, docking experiments were conducted against TLR4 and TLR3 to evaluate the interaction of secondary and tertiary peptides with TLRs, and molecular dynamics simulations were utilized to verify the stability of the vaccine [88]. Thus, bioinformatic tools are effective in predicting mRNA sequences with the potential to be later translated into real-life constructs with optimal efficacy.

## 4. mRNA Vaccines and Adaptive Immune Responses

### 4.1. Role of mRNA Construct Modifications on Adaptive Immune Mechanisms

Many studies have focused on the incitement of specific adaptive immune responses post the co-administration of mRNA constructs in an LNP nanocarrier vehicle. Administration of an LNP-encapsulated modified NRM vaccine construct against influenza H10 HA was shown to activate H10-specific CD4 T cells confined to the lymph nodes that drained the sites where the vaccine was administered [64]. Moreover, usage of mRNA encoding tumor antigens with different degrees of N1-methylpseudouridine (m1Ψ) modification was examined for immunogenicity and anti-tumor responses in a B16 melanoma model [65]. It was shown that unmodified mRNA constructs were more useful in improving melanoma conditions compared to the nucleotide modified type, and inhibition of type I IFN receptors entirely reversed the anti-tumor immune response elicited by unmodified mRNA. This reversal was evident in a notable reduction in T cells secreting IFN-γ and an increase in the presence of programmed cell death protein 1 (PD-1)+ T cells infiltrating the tumor [65]. Moreover, LNP-encapsulated mRNA vaccines against HCV were conjugated with viral ectodomains of E1, E2, or a modified E2 with reduced CD81 binding and an inserted N-linked glycosylation site. When a soluble E2 was modified and added to the vaccine construct, there was elevated neutralizing antibody responses, an increase in total IgG production, and further isotype switches [67]. Thus, nucleotide modifications in mRNA vaccines can incite considerable adaptive immune responses, beneficial in combating disease outcomes.

### 4.2. Role of TLR Agonism in mRNA Vaccination on Adaptive Immunity

TLR agonism has been shown to have adjuvanticity functions leading to a more robust adaptive immune response. The study by Pan and colleagues showed a significant increase in the activation markers of CD69 in B cells, CD4+ T cells, and CD8+ T cells after inoculation with an anti-tumor vaccine co-loaded with sLNPs, OVA-coding mRNA, and TLR4 ligand MPLA in a prophylactic mouse model, demonstrating the effectiveness of adaptive immune enhancement with TLR4 agonism (Figure 2B) [80]. sLNPs-OVA/MPLA generated the highest antigen-specific CD8+ T-cell response, and the most significant blood OVA-specific IgG antibody titers compared to a control (Figure 2B) [80]. Moreover, an mRNA vaccine developed against the RBD of SARS-CoV-2 used charge-altering releasable transporters as a delivery vehicle, and showed that the addition of CpG, a TLR9 ligand, demonstrated anti-RBD-specific antibodies at day 4 post immunization, as well as the early isotype switching of these antibodies (Figure 2C) [82]. In addition, the same CpG-adjuvanted vaccine induced memory Th1 CD4+ and CD8+ T-cell responses. This vaccine formulation further led to Th1 polarization in vitro, as the lung cells of adjuvant-vaccinated mice restimulated with the RBD protein expressed CD4+/CD44high/TNF-α+ (Figure 2C) [82]. Thus, TLR agonism can effectively alter lymphocyte infiltration and populations post mRNA vaccine inoculation.

### 4.3. Role of Encapsulation Material on Adaptive Immunity—An Outlook on Chitosan

Chitosan nanoparticle encapsulation has been shown to play a role in adaptive immunity. Oral inoculation with a chitosan-encapsulated mRNA vaccine made against the outer membrane protein and flagellin of SE led to increased proliferation of splenocytes of broiler chickens at days 7 and 21 post vaccination, as well as higher mucosal IgA levels after receiving 50 ug of the chitosan-coated mRNA vaccine [74]. The same group of researchers investigated the same vaccine composition conjugated with mannose instead and found an increase in the infiltration of cytotoxic T cells in the mannose-only conjugated group, as well as upregulation in γδ T-cell frequency in the group co-conjugated with the mannose and flagellin of SE [75]. The molecular weight of chitosan particles was also shown to impact adaptive immunogenicity in vitro—LMW and HMW chitosan particles were examined for their effects in BMDCs [77]. HMW chitosan treatment at 10 µg/mL led to a significant increase in the infiltration of CD80+ and CD86+ as well as the elevated expression of major histocompatibility complex (MHC) class II [77]. Moreover, LMW chitosan adjuvanticity was shown to enhance IgG production after vaccination against HA molecule of Influenza A virus in mice. Both LMW and HMW chitosan-coated vaccines led to increased CD69+/CD103+ CD4 and CD8 T-cell populations in the lung post viral challenge. Nonetheless, only HMW chitosan-coated vaccine led to an increase in the infiltration of IL-2-producing CD44+/CD4+ T cells in the draining lymph nodes 2 days post challenge [77]. Recently, a recombinant VRTPIL-7 plasmid, encapsulated with chitosan nanoparticles, and conjugated against a rabies vaccine construct in mice, led to an increase in CD4+ and CD8+ populations as well as an increase in neutralizing antibodies and IgG-specific responses [78]. Moreover, conjugation of a chitosan-coated vaccine against a swine influenza virus antigen with TLR3 ligand, poly(I:C), was shown to significantly upregulate infiltration of IFN-γ-secreting T-helper cells, while chitosan coating alone led to an increase in γδ T-cell frequency in PBMCs isolated at day 6 post challenge with H1N2-OH10 virus [79]. Therefore, the chitosan encapsulation of mRNA has shown to induce considerable adaptive immune responses post inoculation.

## 5. Concluding Remarks

Exploring the interplay between innate sensing mechanisms and adaptive immune responses post mRNA vaccination has revealed avenues for enhancing vaccine efficacy. mRNA-based vaccines harness the host’s innate immunity to trigger robust adaptive responses against various pathogens. Understanding how mRNA vaccines engage with the innate immune system aids in tailoring vaccine designs for optimized immunogenicity and safety. Modifications to mRNA constructs or encapsulation material significantly impact innate sensing and subsequent immune responses, with further alterations, including innate adjuvants, optimizing the vaccine platform. Leveraging this knowledge to fine-tune mRNA vaccine formulations, delivery systems, and adjuvant strategies is crucial for progress. Balancing immune activation for protection while minimizing adverse effects is a key goal in mRNA vaccine development. Continued refinement in this area holds promise in combating infectious threats and advancing vaccination technology.
